# *Exit*, *Voice*, and *Loyalty* in the Italian Public Health Service: Macroeconomic and Corporate Implications

**DOI:** 10.1155/2013/292745

**Published:** 2013-11-21

**Authors:** Adelaide Ippolito, Cira Impagliazzo, Paola Zoccoli

**Affiliations:** ^1^Research and Development Board, Federico II University Hospital, Via Sergio Pansini 5, 80131 Naples, Italy; ^2^Department of Studies and Business Research, University of Salerno, Via Giovanni Paolo II 132, 84084 Fisciano Salerno, Italy

## Abstract

The paper analyses how customers of public health organizations can express their dissatisfaction for the services offered to them. The main aim is to evaluate the effects that possible dissatisfaction of Italian public health service customers can have on public health organizations. We adopted the methodological scheme developed by Hirschman with *exit, voice,* and *loyalty*, considering the macroeconomic and corporate implications that it causes for Italian public health organizations. The study investigated the effects developed by exit of the patients on the system of financing of local health authorities considering both the corporate level of analysis and the macroeconomic level. As a result, local health authority management is encouraged to pay greater attention to the *exit* phenomena through the adoption of tools that promote loyalty, such as the promotion of voice, even if *exit* is not promoting, at a macroeconomic level, considerable attention to this phenomenon.

## 1. Introduction

In private and public sectors customers can express their disappointment with goods or services produced by a particular company through two main types of actions: *exit* or *voice*, studied in great detail by Hirschman [1].

*Exit* is the classic option for the customer, with it being typical of market systems where there is free competition (economists consider it an efficient alternative and have dedicated more attention to it than the other option). It occurs with the customer's moving away from the economic or social organization owing to dissatisfaction, and it does not necessarily imply the transition from public to private sector and vice versa [2]. The presence of a high number of alternatives, as expected in situations of perfect competition, actually makes this option easily practicable by the customer. The progressive reduction in the number of customers, as highlighted by a reduction in sales, will lead the organization to take action in order to correct the causes of dissatisfaction otherwise the organization will be doomed to extinction [1]. Subsequently, Mizrahi [3] and Gofen [4] have conceptualized, in the context of public services, an entrepreneurial exit referring to citizens who exit proactively by creating a viable alternative themselves.

*Voice* is the distinctive option, though not exclusively in politics, but economic frameworks as well, in which there is either a monopoly system in force or few alternatives on offer. Regarding *voice*, Dowding et al. [5] made a distinction between individual *voice* and collective *voice*, while Light et al. [6] developed the concept of organized *voice*. Regarding the concept of collective *voice* in health services, many of its advocates consider it a tool that can make healthcare organizations and providers more responsive [7]. *Voice* takes place through the communication or rather the protest (it, in particular, can have a rather general connotation from the simple complaint to the violent protest) by the customer of those situations of dissatisfaction, which are due to the organization. It does not cause the suspension of the established relationship, provided that an active commitment of the organization to the elimination of the causes of dissatisfaction follows *voice* [1].

Both options (*exit* and *voice*) can work in either a combined or alternate way with each other [5, 8], with it being obvious that the accessibility or the effectiveness of these ways varies according to the situations which can arise. The use of *exit* depends on the availability of alternatives which are comparable for the customer (for an analysis of the intergroup differences in the *exit* propensity see Tai-Seale [9], while for an analysis of the effects of the introduction of choice in UK health services see Greener [2], Fotaki [10], and Peckham et al. [11]), more particularly,

- on the existence or not of these alternatives,
- on the knowledge that the customer has of their existence,
- on their accessibility, meant above all in terms of cost,
- on the qualitative level of the latter.

The effectiveness of *exit*, as a warning sign for a company to the occurrence of a decline in quality, depends on various factors. Firstly, the disaffection of customers so that it can give rise to an appropriate corrective action by the company. It should be neither too measured nor too high because if the reduction of customers is minimal, the company will not be pressed for the reaction, while if the reduction of customers is too high, the company has to come out of the market before having given rise to corrective actions. This implies, therefore, the need to maintain the qualitative elasticity of demand at a medium level [1]. Secondly, a qualitative deterioration of the company's products as a general phenomenon should not occur, that is, as a phenomenon which is imputable to all companies of the same sector. In this case, we could actually have ineffective functioning of *exit* if every company of the sector acquired some of the disgruntled customers of other companies even at the cost of losing part of the previous customers in favour of the new candidates [1].

Regarding the *voice* option, its viability depends on the existence of a bond of loyalty between the customer and organization, besides it depends on the effectiveness with which the organization will find a remedy for the causes of dissatisfaction. Of course, this statement is valid for an analysis of a general nature because not all complaining customers are loyal, furthermore; *voice* is also applicable to competitive economic contexts. *Voice* option seems to be the most favourable alternative for the organization; since it does not result in the termination of the relationship with the customer, it allows the organization to rectify its own mistakes without the loss of the customer. Moreover, it is the most plausible alternative in situations characterized by a low elasticity of demand or by conditions that hinder the *exit* [1]. However, in some situations, increased voice can create problems; in this regard, the health programs sponsored by the Office of Economic Opportunity [7] showed that the consumer's *voice* and participation can have negative effects on decision-making and on organizational planning.

Even in the case of *voice* option, there are some conditions that influence the effectiveness. In order to ensure, in front of a decline in quality of a company, a reaction of protest and not of disaffection by customers, it is necessary that they are convinced of the effectiveness of *voice* and that, moreover, its cost is not excessive for those customers who decide to protest; in this regard, both for *voice* and for *exit*, van de Bovenkamp et al. [12] analyzed the delegation as a tool that reduces the work that the use of these options require. From this point of view, users are also more inclined to use *voice* for those goods and services that have a significant economic impact, while the inclination to the use of *voice* decreases as the economic impact of those goods and services declines. In a similar manner, Carmel [13] argues for hospitalized patients that dissatisfaction results in a response related to the patients' perceptions of the severity of the service failure and their relative social power. Furthermore, the possibility of using the *voice* can be reinforced if there is the alternative of *exit* [14], although there are contrary opinions [15].

In the economic situations characterized by various conditions of monopoly, as is still partly the case for the sector of public services (although it should be noted that the conditions of monopoly are mainly connected to the service rather than to the satisfaction of needs), users can show their dissatisfaction with the quality of the services provided primarily through *voice* in the absence of alternatives of *exit*. This should spur the management of public utilities to improve the quality of the services provided. From a theoretical point of view, the increase in the alternatives tied to the introduction of conditions of competition causes the increase in the adoption of *exit* option by customers to the occurrence of declines in quality of services. This condition should encourage the management of public utilities, detecting a reduction in the volume of the services provided, to find a remedy for these qualitative declines. The conditions provided for the analyzed model should, therefore, be valid for the public sector too but, as Hirschman [1] underlines, if the management relied on financial resources which are independent of the extent of the services provided (such as the automatic balancing of the budget's deficit), the increase in conditions of competition, promoting the phenomena of *exit* as to the use of *voice*, could make the management insensitive to the reduction in the volume of the services provided as a warning sign of a qualitative decline in services.

The concept of *loyalty* is unlike the concepts of *exit* and *voice,* not adequately determined, with it being presented as a construct which acts when customers have recourse to the option of *voice* although they have at their disposal the alternative of *exit* [1, 5, 16]. However, according to some, the *loyalty* option is connected to an attitude of optimistic expectation of improvement [17, 18]. The *loyalty* option is presented as the product of a number of factors that join the user to the organization, making it little practicable or expensive in some contexts unless there is the expectation of improvement [19], especially in those of a social nature such as the family, the membership of a church, and a political party.

Subsequently, Rusbult et al. [17] developed another form of response to dissatisfaction: *neglect*, which is a passive behavior that indicates a lack of commitment and development of the user as well as a negative stance. Furthermore, *neglect* and *loyalty* are defined as passive responses to the dissatisfaction, while *exit* and *voice* are defined as active responses to the dissatisfaction [17].

On the basis of what has been previously discussed, the aim of this work is to analyse the macroeconomic and corporate implications that, in the Italian public health service, can cause the adoption of the methodological scheme of *exit*, *voice,* and *loyalty* developed by Hirschman considering the type of tools that, in the Italian public health service, may realize the conditions of *voice* and *loyalty*.

## 2. Materials and Methods

### 2.1. The Macroeconomic Implications of *Exit*, *Voice*, and *Loyalty* in the Italian Public Health Service

*Exit* and *voice* options are mechanisms that have an effect on the public health service too. In Italy a mixed public-private system provides healthcare services for all citizens and residents. The public part is the National Health Service *Servizio Sanitario Nazionale* (SSN) which is organized under the ministry of health and its administration is on a regional basis. local health authorities and public hospitals provide public healthcare services. local health authority is an instrumental entity of the region, which includes the financial organization and management of health services (prevention, treatment, and rehabilitation) at the local level, while the public hospital provides treatment to cure disease patients at the acute stage. In-patient care and general practitioner services are free of charge, but copayments are generally required on pharmaceuticals, diagnostic procedures, and specialist visits. The Italian regional health services are funded by resources from the revenue IRAP (regional tax on productive activities), the additional regional IRPEF (personal income tax), sharing the excise on petrol, and a regional partnership to VAT (value added tax). The revenues of the healthcare providers and the balance of patient mobility are added to these funding sources. The Intesa Stato Regioni (Agreement Government-Regions) approves annually the financing of the different regional health services and each region will take its own measure of apportionment. The financing of the regional health current account is the share of the national health fund assigned to the region minus the balance of interregional patient mobility and the additional contributions from the budget aimed at ensuring regional funding support levels established by the region with the regional health plan. The allotment to providers is made on the basis of the following criteria:

- hospitals for the remuneration rate of the services provided;
- for healthcare substantially the weighted capitation for all levels of care with the exception of prevention, primary care, and community care for which the policy applies to the simple capitation and benefits that each company has secured in previous year and that are required to ensure the new financial year, for the purpose of achieving uniform levels of service.

To apply the analysis of *exit* and *voice* options to the Italian context, it is necessary to separately examine the effects that they have at a macroeconomic level from those that can be produced at a corporate level. 

From a macroeconomic point of view, in the National Health Service, patients may have recourse to those health services which are provided by the private market such as private health organizations operating within the National Health Service (accredited providers) or ones not operating within the National Health Service. In the last OECD report [20] about health expenditure (the data refer to 2010), the nation that registered the highest total health expense is the United States with 17.6 percent of the GDP, almost twice the OECD average, followed by The Netherlands, France, Germany, and Switzerland. Italy ranks the thirteenth, in the middle, with an expenditure of 9.3 percent of the GDP of which about 2 percent is private. In particular, the percentage of private expenditure on the total amount of health expenditure was 17.8 percent in 2010. This last percentage has decreased over the years, from an average of 23.92 percent in the decade 1991–2000 to an average of 20.28 percent in the decade 2001–2010. It, however, is still high in comparison with other countries of the OECD that have public health systems. With the exception of Finland, which in 2010 registered a percentage of resort to private health expenditure of 19.2 percent in comparison with the total amount of expenditure, other countries that registered a percentage of private expenditure that is higher than that of Italy (17.8 percent) are those where the current economic crisis is felt more (Greece, 38.4 percent; Portugal, 26.0 percent; and Spain, 19.7 percent), Ireland has a percentage of private expenditure of 17.4 percent, Sweden of 16.8 percent, Denmark of 13.2 percent, and the UK of 8.9 percent.

 The phenomenon of the considerable resort to the private health expenditure in Italy can be attributed to several factors:

- the economic crisis, which involves a different attitude by families in the resort to the private health expenditure;
- the result of the comparison between levels of effectiveness that are expected to be obtained from private health organizations and those of public and private health organizations operating within the National Health Service, which work as providers of the public health system;
- the result of the comparison between the different waiting times required for the access to the health service;
- the result of the comparison between the two different levels of caring and accommodating comfort.

Among the aforementioned factors that encourage the growth of private health expenditure, the comparison between the different waiting times required for the access to the health service plays a main role. In this regard, in a survey sample carried out in Italy in 2010 [21] it was shown that the waiting lists most suffered by the citizens are the diagnostic tests (52.6%) followed by first access of specialist visits with 28.2%, while complaints for long waiting times of surgery are under 20%. Oncology is the one that suffers the most of the expectations of the diagnostic tests (18.2%), followed by the gynecology/obstetrics (about 16%) and cardiology (14.4%). In particular, ultrasound (8 months), nuclear magnetic resonance or computerised tomography (10 months), and mammography (12 months) are the tests whose reports indicate longer times. Regarding medical examinations it is the ophthalmology that most reported for delays (19.7%) followed by cardiology (10.1%) and dentistry (10.1%).

Although in recent years there has been constant resort (disaffection) to the services delivered by private health providers, the number of people contributing to the financing of the national health system does not change; the patient who benefits from private health services is, however, obliged to contribute (with his income) towards public health expenditure through taxation. This would not contribute, applying the analytical scheme developed by Hirschman, to the stimulation of the National Health Service in the development of processes for the improvement in health services provided in the public sector, which could contribute to the reduction in disaffection. On the contrary, the Italian government would tend to encourage this phenomenon allowing, within certain limits, the deductibility from the taxable income of such expenses, both those related to the attainment of single services and those related to the taking out of private health insurances, consequently, weakening the ability to effectively exercise the other option, namely, protest (*voice*).

The creation of a situation of competition in the public healthcare sector cannot, therefore, have positive effects, mainly due to the lack of effective incentives compared to those which work in the private sector for public health workers, who often are unable to react against the phenomena of *exit*. Moreover, the increase in the situations promoting *exit* has had, in its turn, negative effects on the effectiveness of the functioning of the protest.

## 3. Results and Discussion

### 3.1. The Corporate Implications of *Exit*, *Voice*, and *Loyalty* in the Italian Public Health Service

From a corporate point of view, all the effects that the two mechanisms of *exit* and *voice* develop for the single health providers operating in the public healthcare sector must be examined. It is worth considering that, in this work, preference will be given to the effects on the economic balance of public health organizations rather than to the social and ethical implications. The balance effects are able to alert for future social effects.

Although the following observations are valid, even if within certain limits, for all those providers who work as suppliers of the Italian National Health Service (NHS), that is, both public and private health organizations operating within the NHS, the analysis will be limited to the local health authorities because they represent a more complete field of research. We will try, in particular, to demonstrate how the local health authorities (in Italy ASL) cannot remain indifferent to the phenomena of disaffection or protest by users who belong to the territorial area which falls within their competence.

It is appropriate, in this context, to recall that the local health authorities are financed by the regional health fund and the criteria used vary according to the different regional situations. In particular, an interesting aspect of *exit* for local health authorities is the mobility of customers between public or accredited providers belonging to different regions. Customer mobility in the NHS, in fact, is a phenomenon of critical issues in Italy, as it involves about 860,000 citizens every year with a value, in 2011, of about 3.8 billion Euros. An analysis (mondosanita.it) shows that it is mainly the regions of Southern Italy that have a strong passive mobility, which is especially pronounced in the regions with the highest public debt such as Campania (in 2011, it had a negative balance for health mobility of 311 million euros), Calabria (in 2011, it had a negative balance for health mobility of 238 million Euros), Sicily (in 2011, it had a negative balance for health mobility of 197 million Euros), and Puglia (in 2011, it had a negative balance for health mobility of 177 million Euros). Conversely, the regions of Northern Italy frequently have an active mobility, such as Lombardy (in 2011 it had a balance for mobility health of 492 million Euros), Emilia Romagna (in 2011, it had a balance for health mobility of 342 million Euros), Tuscany (in 2011, it had a balance of mobility health of 121 million Euros), and Veneto (in 2011, it had a balance of mobility health of 87 million Euros). With reference to the analysis of exit, if a customer resorts to a health provider different from that managed by his own local health authority, in order to satisfy his/her own needs of getting a health service, it is necessary, as a result, to take all those effects into consideration, which result from different situations:

1. *the health service cost is entirely borne by the customer* (the services to which reference is made are, e.g., microbiological tests, a large part of the medical imaging, outpatient visits, and so on; if they are not paid to weak segments of the population, which benefit from the tax exemption, they are completely at the user's expense, i.e., services with charges). In this case, the local health authority is not indifferent to *exit* because it has lost the opportunity to provide a health service losing the possibility of contributing to the covering of its own fixed expenses and also to the formation of an additional margin (in a general analysis);
2. *the health service cost is partly paid for by the National Health Service and partly paid by the customer (copayment)*; in this event we can have two further situations:
  - the customer satisfies his needs in a private health organization not operating within the NHS. In this situation, the economic advantage of the local health authority does not change, even if it loses the share of additional resources charged to the customer (copayment) and connected to the loss of the service;
  - the customer satisfies his needs in a public health organization different from the local health authority he/she belongs to or in a private health organization operating within the NHS (accredited provider). In this situation, the local health authority, not directly supplying the service, loses the share of resources at national health system expense, which is necessary to restore the cost paid by the customer and of course the amount paid by them (copayment);
3. *the health service cost is entirely paid by the NHS (services free of charges)*; also in this case there are two possible situations:
  - the customer gets his/her service in a private health organization not operating within the NHS; in this situation the economic advantage of the local health authority does not change (even if it does not provide the service, it will not lose the share of resources related to its cost). On the contrary, according to the viewpoint of the mere economic advantage, if the marginal cost borne to provide the service was higher than the marginal value of the cost established, this could be advantageous for the local health authority;
  - the customer goes to a public health organization different from the local health authority he/she belongs to or to a private health provider operating within the NHS (accredited provider); in this case, the local health authority loses the share of resources related to the total cost of the service.

The different analysed situations deserve clarification because if the local health authority maintains an effective cost accounting based on a cost centre architecture, which allows it to do a comparative analysis of costs, results, and returns, the management may decide to differentiate its policies aiming at reducing the phenomena of *exit*. Indeed, the analysis may consider whether the tariffs of the services provided are more or less profitable to the respective health services costs incurred by the local health authority or whether the local health authority is not yet able to provide such services under conditions of productive efficiency. Thus, the local health authority policy could be aimed at avoiding the phenomena of *exit* for those services with profitable costs and at facilitating the phenomena of *exit* for the services whose rates are not profitable.

The previously expressed considerations on the economic effects of the *exit* phenomena on local health authorities are, however, to be regarded as purely theoretical in regional frameworks where there is not a real condition of competition in the healthcare sector. In many regions of Southern and Central Italy, there is a financing system for public hospitals and local health authorities where there is not a change in the traditional model, which is previous to the beginning of the process of business management that still depends on the historic cost. Furthermore, the control of health expenditure is implemented through the prediction of expenditure ceilings.

Under these conditions, the increase in customer *exit* in favour of the private sector (operating within the NHS or not) does not necessarily bring about a tendency of the management to the improvement in the services provided. In fact, if the local health authority does not work under conditions of efficiency, the management, being funded on the basis of the historic cost, will not be effectively accountable for the qualitative level of the services provided (the *exit* of customers could not produce incentives to the change either). On the other hand, if the public health authority works under conditions of efficiency, the management can paradoxically consider the phenomena of customer *exit* as desirable, if the amount of services provided exceeds the value of the historic cost or, however, is not very sensitive to such *exit*.

In the case of absence of economic incentives related to the *exit* phenomena, the social and ethical requests, which must be the basis of the management's behaviour in local health authorities, are of crucial prominence. In this situation, the continuous attention to the task and values that characterize these authorities is even more important with them being the only incentive for an improvement in the quality of the services provided and to give the due interest to the incentives that come from customers.

**Figure 1:**
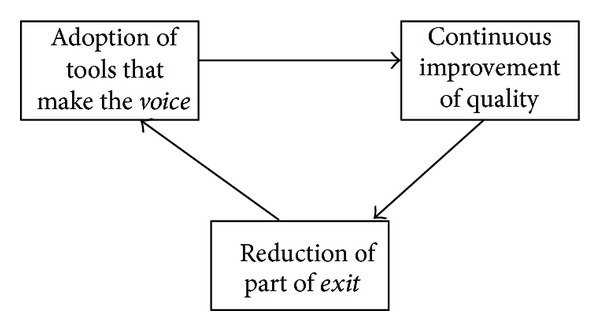
Factors that allow the health organization to reduce part of *exit*'s phenomena.

Local health authority must therefore necessarily try to restrain the *exit* phenomena; otherwise it will suffer a progressive loss of the resources at its own disposal. Since a part of this *exit* may be due to the poor quality of the services provided, local health authority should take (for the specific *exit* phenomena) suitable measures in order to develop and make effective the tools aiming at ensuring the voice, which allow the local health authority to foresee the occurrence and the causes of the situations of reduction in the level of quality of its own services and at the same time the development of a process of continuous improvement in the latter (Figure 1). It would not have meaning for the local health authority to adopt an effective system for monitoring the level of satisfaction of its own services, if the same system did not bring about a rapid and real improvement in the services provided.

It must be pointed out, however, that the arrangement and effectiveness of the actions aimed at reducing defection are related to the degree of sensibility to the problem shown by local health authority management. In particular, there might be three types of behaviour that are consistent with the type of management style, which can be bureaucratic, managerial, or entrepreneurial:

- passive: the management of local health authority does not react against the phenomena of *exit* of its own customers;
- reactive: management of local health authority only reacts against those phenomena of *exit* which are related to losses of resources, originally addressed to public health providers and to private health providers operating within the NHS;
- proactive: management of local health authority not only reacts against the loss of resources in favour of public health providers and private health providers operating within the NHS, but also spares no effort to acquire further resources trying to develop the selling of health services whose cost is borne by the customer.

**Figure 2:**
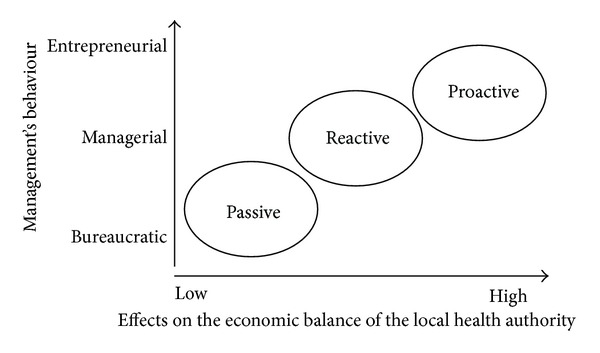
Effects on the economic character of different behaviours of the local health authority's management.

It is obvious that the adoption of a proactive approach has positive effects on the economic balance of the local health authority (Figure 2); it allows a more efficient exploitation of the productive capacity and positively influences the image of local health authority.

### 3.2. The Tools Promoting *Loyalty* and *Voice* of the Italian Public Health Service Customers

The Italian public health organizations, and within this above all the local health authorities, have the need to limit the phenomena of customer *exit*. This is a deeply felt need since, in recent years, some rules have, explicitly or implicitly, favoured the exercise of the right to the freedom of choice, that is, the right that allows customers to choose between public and private health organizations operating within the NHS providing health services; the most appropriate health organizations which are to satisfy their own needs. Only with law 833/78 (article 19, paragraph 2) was there a first formal recognition of the right to the freedom of choice in the Italian public health service. Subsequently, this right became effective with the legislative decree 502/92 (art. 8 and art. 14, paragraph 6), the directive of the prime minister on January 27th, 1994, and the decree of the prime minister on May 19th, 1995.

We must, however, specify that the actual possibility to exercise the free choice gives prominence to the reasons adopted by the customers in the choice of the health organization to which the users turn. In particular, there is a difference between the overriding criteria which customers tend to follow in order to make their choices (in particular the expertise of physicians, the equipment, the health organization's closeness to the customer's residence, the kindness and the humanity of the staff, the comfort of the health organization, and the possibility to schedule the dates of stay) and those that effectively affect the concrete choice (identified as follows: the doctor's advice, the presence of the customer's doctor in the health organization, the closeness of the health organization to the customer's residence, experiences of previous hospitalizations in the same health organization, information or advices given by their own family and friends, and the expertise of physicians).

Since the reasons why customers choose the health organization to turn to are complex, the analysis of the developable actions to reduce the *exit* phenomena will be focused, as previously stated, on those which, from a marketing point of view, are linked to the qualitative level of the services. According to this argument, public health organizations should have as an overriding aim the development of policies aimed at promoting and facilitating the manifestation of the *voice* phenomena by customers so that the customers do not actually give rise to the *exit* processes due to the occurrence of the phenomena of disservice, but express, however, their dissatisfaction. Public health organizations must encourage the development of policies aimed at facilitating the *voice*, which take part in the creation of processes of communication with the customers, or boost the promotion of a bond of trust and loyalty with its own customers. The more the health organizations develop a bond of *loyalty* with their customers, the more unlikely they turn to other health organizations to benefit from health services due to the occurrence of cases of dissatisfaction.

The need to promote a bond of *loyalty* with the customers is particularly felt in the health services that, being aimed at the satisfaction of people's health needs, have their essential component in the creation of relationships based on trust and loyalty. So as to realize this bond of *loyalty*, public health organizations must adopt a management focused on quality, that is, on the continuous improvement in health services provided, through the identification of customers' needs and their satisfaction in the most effective possible way. This requires an active involvement of all human resources of the public health organization in order to attain this goal together with the promotion of a more informed, active, and aware relationship with the customers.

The adoption of a management focused on quality can be encouraged by the use of different tools. From a marketing point of view, it could be fostered by the development of a process of empowerment [22, 23], which is both internal and external.

Internal empowerment is based on a process of decision-making delegation in favor of health workers and, conversely, of accountability of the obtained results. It is essential to ensure the participation and the sharing of health workers in qualitative business aims, through human resources' policies which aim at assuring workers of their own incitement. So workers can continuously consider themselves as part of a relationship between the user and the provider which forms a chain that extends across the public health organization and ends with the final customer. In this case, empowerment has its source in a process that activates positive conditions in the self-operating of employees. This generates conditions to build the relationship with the user by the worker to activate the interaction at the base of the response to the health need.

External empowerment refers to the process of empowerment and participation of customers that in recent years has been implemented in the public health service. External empowerment, deriving from the condition of the context of policy and organization of public health organization to make the citizen and user of health service aware, makes the citizen be part of the purposeful and effective action. In this case, the relationship comes from interaction in the institutional context.

The customers never accept in their contacts with the public health organizations the position of being inactive, but they are aware of the possibility of becoming active participants in defence of their own health. This is achieved through the adoption of policies, in particular public, aiming at the production and diffusion of information of quality addressed to citizens in order to allow them to look after their health in the most independent way possible, to have recourse to health services with full knowledge of the facts, and to benefit from the services provided in a more conscious way. The above features are therefore perfectly combined with the public need to promote in customers more rational and responsible “health consumption,” aiming not only at the control of the health expenditure but also at a more conscious view of the actually expected effectiveness or, rather hoped, of health services, so as to strive for a greater equity and efficiency of the health system and for a satisfaction of customers' needs. This new customer role, on the one hand, gives them more rights and discretionary areas while, on the other hand, it requires a more participating and responsible behavior in the process of services' provision from them encouraging by contrast their satisfaction.

The public health organizations' choice of adopting a management focused on quality contributes to positively affecting the image of the organization itself on the market, even if it is largely influenced by the level of complexity and specialization of the services offered by the public health organizations. The result of this process contributes to the development of customer *loyalty* towards their own health organizations.

With regard to the implementation of policies aimed at promoting *loyalty*, public health organizations must facilitate the manifestation of the *voice* phenomena not only to the occurrence of cases of inefficiency, but also in all the situations where it is necessary for the public health organization to develop communication flows through the planning and management of special tools that directly or indirectly support this need.

The tools at the Italian public health organizations' disposal for promoting the implementation of *voice*, meant as an interactive process of communication between customers and public organizations themselves, are the public relations office, the active management of complaints, and the tribunal for patient rights.

The public relations office (in Italian URP: “Ufficio per le Relazioni con il Pubblico”) was established by article 12 of the legislative decree number 29 on February 3rd, 1993, concerning “the rationalization of the organization of civil services and the revision of rules and regulations on the civil service, in accordance with article 2 of the law of the 23rd of October, 1992, number 241” and later regulated by the directive of the prime minister on October 11th, 1994 called “directive on the principles for the establishment and the perfect running of public relations offices.” 

It should be pointed out that the public relations office not only plays the role of a tool for the listening of complaints that the customers can make towards the services provided and of the suggestions that they can give to improve on the services, but also plays a real role of “trait-de-union” between public utilities and customers. It realizes, in addition to the listening to complaints, the customers' reception, communication, and information to the customers, also beyond the incentives developed by them and the marketing functions for the propulsive and organizational role played in public utilities.

A further tool of *voice* at the health public organizations' disposal is the active management of complaints. The complaint is actually the typical means of expression of user dissatisfaction not only in the public health service, but also in different socioeconomic frameworks. Even if it is related to a negative experience for the customer, it is a very important tool for understanding and identifying the situations of inefficiency and the possible fields of improvement of goods or services by public health organizations. In addition, the complaint can be an effective means to obtain customer *loyalty*, if it is properly managed through the recovery, that is, the set of actions that the public health organization has to perform in order to correct a previous error and get the customer back.

Handling complaints in the public health service and in general in the public sector in a proper way requires a cultural action which goes beyond the simple arrangement of physical places and appropriate tools for the collection of complaints but entails the inclination to the listening to situations of discomfort experienced by users. The aim of this action is therefore to press customers for the manifestation of the discomfort phenomena and to give rapid and effective replies, not only with regard to the single problems that occurred, but also with regard to the actual improvement processes in the service provided. Only in this way will a climate of trust between customers and public health organizations develop together with the consequent *loyalty*, which will encourage the communication of further situations of discomfort that may occur; otherwise, if an actual improvement in the service provided does not follow the complaint, there will be a feeling of mistrust, which will stimulate the actions of *exit* from the public health organizations.

This implies, therefore, the need not only to organize the instrumental and human resources necessary for the collection of complaints in an appropriate way (special office, telephone, fax, toll-free number, complaint box, voluntary organizations, workers, and so on), but also to involve and motivate all the health workers, starting from the management, so as to ensure the due importance to the crucial role attached to the complaints for the improvement in the services provided.

Another tool of *voice* in the Italian healthcare sector is the Tribunal for Patient Rights even if, it should be pointed out, it is not a means that is particular to the health organizations, but only the result of the voluntary work. In particular, the Tribunal for Patient Rights was established in 1980 by the Democratic Federative Movement and has long been one of the main tools to give *voice* to complaints and protect citizens from cases of dysfunctional national health service and situations of denial of rights, as well as squandering and inefficiency which can occur in the health organizations. It works with the voluntary collaboration of ordinary citizens as well as some workers of the national health sector.

The Tribunal for Patient Rights presents itself not only as a very present service in health organizations, but also above all as a very much appreciated and deep-rooted service in user experiences, so, even if it is not a tool that is peculiar to public health organizations, they can actively cooperate with it in order to identify any disservice phenomena.

## 4. Conclusions

The previous analyses underlined the macroeconomic and corporate implications which, in the sector of the Italian public health service, may cause the adoption of the methodological scheme of *exit*, *voice,* and *loyalty* developed by Hirschman.

The application of the methodological scheme developed by Hirschman to the Italian public health service stressed, from a macroeconomic point of view, that the creation of a situation of competition does not have any positive effects due to the lack of effective incentives compared to those that generally are in the private sector for public health workers, who can not often react against the *exit* phenomena. Different assessments are related to the corporate point of view of the methodological scheme developed by Hirschman. Local health authority must try to restrain the *exit* phenomena; otherwise it will suffer a progressive loss of the resources at its own disposal, above all through the adoption of those tools that promote *voice* and *loyalty*.

As previously stated, the management of Italian public health service should give more attention to the phenomena of customer *exit* and adopt policies aimed at encouraging the creation of bonds of *loyalty* with the customers themselves, through the promotion of tools that aid the *voice*. 

This paper confronts the issue from a purely theoretical point of view, but further analyses can be developed so as to empirically verify such statements. In addition, our analysis has only considered situations of *exit* related to the quality of health services provided; in fact, there may be phenomena of *exit* due to a lack of supply capacity, which would require management interventions that can increase the offer of health services in order to create a redundancy in supply structures. Moreover, even in situations of *exit* related to the quality of health services provided, the public health service management may consider it appropriate not to reduce *exit* in some specialty care, but choose to focus on the types of health services that could attract customers.
